# Comparisons of Factors Influencing Cancer Rescreening Intention in Middle-Aged and Elderly

**DOI:** 10.31557/APJCP.2021.22.8.2521

**Published:** 2021-08

**Authors:** Hee-Jung Kim

**Affiliations:** *College of Nursing, Research Institute of Nursing Science, Daegu Catholic University, Republic of Korea. *

**Keywords:** Belief, cancer screening, elderly, middle-aged

## Abstract

**Objective:**

This study was conducted to identify and compare factors that influence the intention of middle-aged and elderly in Korea to undergo cancer screening.

**Methods:**

The participants were 367 middle-aged individuals aged 40 to 59 and elderly older than 60 years who had been undergoing cancer screening in D-city, Korea. Data were collected using structured questionnaires that asked about knowledge, health beliefs, and satisfaction regarding cancer screening as well as cancer re-screening intention. The data were analyzed using the SPSS/WIN 25.0 program.

**Results:**

Factors influencing cancer re-screening intentions differed between middle-aged and elderly populations. The influencing factors of cancer re-screening intention among middle-aged were perceived benefits, perceived barriers, and perceived severity of cancer screening, while among the elderly perceived benefits, experience with cancer screening, and the reason for cancer screening were identified.

**Conclusions:**

The results indicate that a customized and systemic approach considering the characteristics of middle-aged and elderly is needed to promote consistent cancer screening in middle-aged and elderly.

## Introduction

The average lifespan has been greatly extended owing to the development of medical technology and improvement of living conditions. In 2019, life expectancy was 83.3 years; elderly persons accounted for 16.5% of the total population, with Korea having the most rapidly aging population among countries part of the Organisation for Economic Co-operation and Development (OECD). Cancer is the most common cause of death among the elderly (Korean Statistical Information Service, 2021). In Korea, cancer incidence is reported to be highest among those aged 65–84 years (Ministry of Health and Welfare, 2019). Recently, the number of elderly cancer patients has been rapidly increasing as the elderly population grows with an extended average lifespan. Cancer incidence among the elderly is also rapidly increasing, and the risk of cancer and mortality increases with age (Alibhai and Horgan, 2011). 

Cancer screening is known to be the most effective method to prevent cancer and increase the survival rate (Lee et al., 2014), and the World Health Organization (WHO) recommends cancer screening as the most effective method to reduce cancer incidence and deaths while improving the quality of life of cancer patients (World Health Organization, 2002). In Korea, the cancer screening rate by age gradually increased from 40 to 60 years and gradually decreased after age 60 (National Health Insurance Service, 2020). Cancer screening is critical and is being emphasized because cancer in middle-aged persons greatly hinders the productivity of families and communities and is accompanied by disabilities in social roles. Old age is a period of great vulnerability to disease, along with physical aging, and is accompanied by various health problems. Therefore, it is crucial to develop strategies to increase the rate of cancer screening for elderly and middle-aged adults, and appropriate strategies must be tailored for each age group.

There is a difference between the elderly and adults because they do not have cancer screenings. According to the National Cancer Center in 2018, the number of elderly who responded, “Because I am healthy” ranged from 59% to 62.7%, which was higher than 51.8% for those in their 50s; 4.7% said they had never heard of screening, which was higher than the overall average of 1.7%. The response “Lack of time” was the second most frequent response among those in their 30s to 50s but the seventh most frequent for those in their 70s, indicating that those over age 65 and middle-aged adults differed in their reasons for cancer screening (National Cancer Center, 2018). Berkowitz et al., (2008) also reported the lack of knowledge and of recommendations by physicians as reasons for the elderly to miss cancer screenings, with the former becoming more serious with increasing age. Based on these studies, it is expected that the cancer screening rate and related factors would differ between average adults and the elderly.

Previous studies have reported the following as factors influencing cancer screening: age, education level, exercise habits, health behaviors such as quitting smoking, and perceived benefits or perceived barriers among health beliefs (Kim, 2015; Lee, 2019). A literature review on the factors influencing cancer screening behaviors failed to show consistent results for demographic factors, but consistent results were reported on the perceived benefits or barriers among health beliefs that influenced cancer screening behaviors. However, most of these studies investigated factors influencing cancer screening behavior in adults, and studies on factors affecting cancer screening among the elderly are insufficient. Given that continuously performing cancer screening is effective, it is essential to develop a strategy to increase and maintain the cancer screening rate. Studies have found that cancer re-screenings are also affected by the quality of service and level of satisfaction with the screening institutions (Vedel et al., 2011; Kim, 2015a).

In this study, the author attempted to explain cancer screening behavior by applying the Anderson and health belief models. The Anderson model is a typical perspective that explains the use of medical services by individuals (Anderson, 1995); it is useful in identifying predictive factors for medical service use by the elderly since it classifies the factors into three—predisposing, enabling, and need factors—to consider demographic, sociocultural, and economic variables (Lee, 2018). Meanwhile, the health belief model is widely used in the field of preventive health behavior research to explain the changes in and the maintenance of health behaviors and is explained in terms of four components: perceived susceptibility, perceived severity, perceived benefits, and perceived barriers (Lee, 2019).

Most studies have focused on adults to confirm the influencing factors, but few have compared middle-aged and elderly . Therefore, this study aims to provide basic data for establishing a cancer screening strategy suitable for middle-aged and elderly persons by identifying and comparing factors that influence the intention to retake cancer screening in middle-aged and older adults based on the Anderson and health belief models from various dimensions.

## Materials and Methods

*Study Design*

This was a descriptive survey aiming to identify re-screening intention among middle-aged and elderly who underwent national cancer screening and to compare and analyze the factors affecting re-screening intention.

*Study Subjects*

The participants were adults middle-aged 40 to 59 years and elderly older than 60 who visited the Health Screening Centers at D University Hospital and general hospitals in D city for cancer screening. Multiple regression analysis was conducted using G*power with 10 factors and a significance level, power, and effect size of .05, 95%, and .15, respectively; the sample number was 344. The number of subjects surveyed was 395, once the dropout rate was considered. Those who understood the objectives of this study and voluntarily participated were included in the study. Out of 395 subjects, the data of 367 were used for analysis, excluding 28 questionnaires that were incomplete or had inadequate responses.

*Measurements*

*1) Subject’s Cancer Screening Related Characteristics*

In this study, we included characteristics related to cancer screening as determined by a literature review. According to the Anderson model, gender, age, education level, and marital status were predisposing factors. Enabling factors were income level, employment, private insurance, awareness of national cancer screening, and cancer screening knowledge. Finally, health status, chronic disease, health behavior, and family members who died of cancer were considered need factors.

*2) Knowledge on Cancer Screening*

A questionnaire developed by Kim (2015b) was used to measure knowledge on cancer screening. The tool consisted of five questions answerable by “yes,” “no,” and “do not know,” with a score of 0 for “incorrect answers” and “do not know” and 1 for “correct answers.” A higher score indicates a higher level of knowledge on national cancer screening. In this study, the reliability was KR-20 (Kuder-Richardson formula 20) =0.55.

*3) Health Beliefs Toward Cancer Screening*

For health beliefs toward cancer screening, a tool developed by Li and Cho (2018) was modified for this study. To increase the content validity of the tool, it was revised after review by two professors from the department of nursing, and only items with a content validity index (CVI) of .80 or higher were selected. The tool consisted of twenty questions, with four each for perceived susceptibility and perceived benefits and six each for perceived severity and perceived barriers. Each question was scored on a 5-point Likert scale ranging from 1 (not at all) to 5 (very much). A higher score indicated a higher belief in cancer screening. Cronbach’s α was 0.83–0.93 in a study by Li and Cho (2018), and 0.71–0.86 in this study. 

*4) Satisfaction with Cancer Screening*

The tool for measuring cancer screening satisfaction was based on a questionnaire developed by Yoon et al., (2009), which was modified for this study through a literature review and validity testing by an expert group. The tool consisted of 13 questions on employee attitude, privacy protection, facilities and environment, time and procedure, cost, result notification, and overall satisfaction. Responses were scored on a 5-point Likert scale, with higher scores indicating a higher level of satisfaction. Cronbach’s α was 0.76 in a study by Yoon et al., (2009) and 0.93 in this study.

*5) Cancer Re-screening Intention*

The tool for measuring cancer re-screening intention was based on questionnaires used in a previous study (Ko, 2012), which were again revised for this study. The measure included seven questions scored on a 5-point Likert scale ranging from 1 (not at all) to 5 (very much), with higher scores indicating higher cancer re-screening intention. The Cronbach’s α in this study was .88. 

*Data Collection Method*

Data collection for this study began after obtaining approval from the ethics committee at D University Hospital, located in D city. After obtaining permission to conduct the study from the health-screening center at D University Hospital and General Hospital, the investigator and research assistant visited and investigated. Before data collection, the survey was conducted after explaining to the participants that they could withdraw their participation at any time if they wanted and that the collected data would only be used for research purposes. Written consent was obtained, ensuring the anonymity and autonomy of the subjects. Participation was voluntary, and informed consent was obtained from all participants. Face-to-face surveys were also conducted. The questionnaires took approximately 15 minutes to complete. 

*Ethical Considerations*

The procedures of this study were approved by the Bioethics Committee (CR-19-068-RES-001-R) of D University Hospital located in D city. Regarding ethical considerations, written consent was obtained prior to the completion of the questionnaire, and the objectives and procedures of the study were explained both verbally and in writing. The following were also ensured: that the participants would remain anonymous, that withdrawal or refusal at any time during the study could be expressed with no disadvantage for doing so, and that the collected data would not be used for purposes other than the study. All the participants were given a small token. 

*Data Analysis*

The collected data were analyzed using IBM SPSS (version 25.0; SPSS Inc., Chicago, IL, USA). The characteristics of the subjects, cancer screening knowledge, health beliefs about cancer screening, cancer screening satisfaction, and re-screening intention were measured using descriptive statistics. The t-test and one-way ANOVA were used to compare rescreening intention according to subject characteristics. The t-test was used to compare cancer screening knowledge, health beliefs regarding cancer screening, cancer screening satisfaction, and re-screening intention, while the correlation among the variables was analyzed using Pearson correlation. A stepwise multiple linear regression analysis was also conducted to identify the factors affecting cancer re-screening intention among middle-aged and elderly. 

## Results

*The Characteristics of the Subjects*

A total of 367 participants completed the questionnaires, with 167 middle-aged and 200 elderly, respectively, and a mean age of 51.2 and 67.7 years for the former and latter, respectively. The demographic characteristics of the participants are presented in Table 1.

The general characteristics revealed a difference in gender (p=0.031), education level (p<0.001), employment (p<0.001), monthly income (p<0.001), and marital status (p<0.001) between the middle-aged and elderly groups. More female participants were recruited from the middle-aged group (71.3%) and the elderly group (60.5%). The middle-aged group had a somewhat higher education level than the elderly group, and often had jobs, and monthly income was also high for the middle-aged group.

Regarding health behaviors and cancer screening-related characteristics of the subjects, there was a difference in exercise (p=0.050), meals (p<0.001), private insurance (p<0.001), and chronic disease (p=0.009) between the middle-aged and elderly groups. For the middle-aged group, 32.3% did not perform any exercise; in the elderly, 29.5% exercised 3 to 4 times a week. In terms of meals, 64.1% of the middle-aged group said that they ate regularly, but 87.5% of the elderly group said so. Among middle-aged adults, 92.8% had private insurance, whereas only 64.0% of elderly were privately insured. Finally, chronic disease was present in 44.3% of middle-aged persons and 58.0% of elderly (Table 1).

*Comparison of Cancer Screening Knowledge, Health Beliefs, Satisfaction, and Re-Screening Intention Between Two Groups*

There was no difference between middle-aged and elderly individuals in the knowledge score on cancer screening with 0.76±0.22 and 0.77±0.21 points, respectively, out of 1. In terms of health beliefs, the perceived severity score was lower among elderly with 3.84±0.74 compared to 3.98±0.64 among middle-aged. Among elderly participants, it was lower (p=.037). As for perceived benefit, scores were lower among the elderly with 4.14±0.46 compared to 4.27±0.58 for the middle-aged (p=0.019). Satisfaction with cancer screening out of a total score of 5 was 4.42±0.56 · in the middle-aged group and 4.27±0.51 among the elderly (p=.011). The re-screening intention out of a total score of 5 was slightly higher among elderly with 3.96±0.60 for the middle-aged participants and 4.08±0.53 for the elderly participants (p=0.029) (Table 2). 

*Cancer Re-screening Intention According to Subject’s Characteristics*

The cancer re-screening intention according to the characteristics of the subjects did not show a statistically significant difference for the middle-aged group. There was no significant difference in cancer re-screening intention according to general characteristics in the elderly group. Cancer re-screening intention according to health behaviors in the elderly showed a difference depending on regular meals, perceived health condition, reason for screening, cancer screening experience, and experience with national health screening. Re-screening intention was higher in those who regularly consumed meals (p=0.011), perceived their health status as unhealthy (p=0.043), were recommended for screening by an expert (p=0.039), and had experience with cancer screening (p=0.004)(Table 1). 

*Correlation Between Subject’s Cancer Re-screening Intention and Various Parameters*

In the correlation test of middle-aged subjects, re-screening intention showed a positive correlation with perceived severity (r=0.168, p=0.030), perceived benefits (r=0.271, p<0.001), and cancer screening satisfaction (r=0.186, p=0.016), and a negative correlation with perceived barriers (r=-0.172, p=0.027). In elderly participants, the re-screening intention was positively correlated with the level of knowledge (r=0.168, p=0.030), perceived severity (r=0.183, p=0.010), perceived benefits (r=0.357, p<0.001), and satisfaction with cancer screening (r=0.244, p=0.001) (Table 3).

*Factors Affecting Cancer Re-screening Intention in Subjects*

Stepwise multiple regression analysis was used to identify the influencing factors of the subjects. When the assumptions of the regression analysis of the two models were tested, both were satisfactory. In the analysis of middle-aged subjects, the factors found to be significant for re-screening intention were perceived benefits (p=0.007), perceived barriers (p=0.010), and perceived severity (p=0.042). The explanatory power of the model was 11.9%. In the analysis of elderly subjects, the factors significantly related to re-screening intention were perceived benefits of cancer re-screening (p<0.001), level of satisfaction (p=0.024), cancer screening experience (p=0.038), and reason for cancer screening (p=0.038). The explanatory power of the model was 19.2% (Table 4).

**Table 1:** Cancer Re-Screening Intention According to the Subjects’ Characteristics

Variables	Classification	Cancer rescreening intention					
		Middle aged (n=167)			Elderly (n=200)		
		N (%)	Mean±SD	*p*	N (%)	Mean±SD	*p*
Sex	Male	48 (28.7)	3.99 ±0.58	0.627	79 (39.5)	4.07 ±0.53	0.889
	Female	119 (71.3)	3.94 ±0.60		121 (60.5)	4.08 ±0.53	
Age (years)	40-49	54 (32.3)	3.98 ±0.52	0.67			0.11
	50-59	113 (67.7)	3.94 ±0.63				
	60-69				132 (66.0)	4.06 ±0.56	
	70–79				65 (32.5)	4.09 ±0.45	
	80≤				3 (1.5)	4.71 ±0.49	
Education	Elementary school	3 (1.8)	4.19 ±0.59	0.946	62 (31.0)	4.15 ±0.48	0.089
	Middle school	27 (16.2)	3.96 ±0.47		43 (21.5)	4.06 ±0.55	
	High school	63 (37.7)	3.96 ±0.58		62 (31.0)	4.05 ±0.55	
	College	60 (35.9)	3.94 ±0.69		29 (14.5)	3.94 ±0.50	
	Graduate school	14 (8.4)	3.87 ±0.47		4 (2.0)	4.64 ±0.44	
Employment	No	48 (28.7)	4.03 ±0.65	0.272	123 (61.5)	4.09 ±0.49	0.802
	Yes	119 (71.3)	3.92 ±0.57		77 (38.5)	4.07 ±0.58	
Smoking	No	130 (77.8)	3.96 ±0.61	0.325	169 (84.5)	4.09 ±0.50	0.563
	Smoking cessation	18 (10.8)	4.07 ±0.47		20 (10.0)	4.06 ±0.71	
	Yes	19 (11.4)	3.78 ±0.56		11 (5.5)	3.92 ±0.51	
Drinking (per week)	No	83 (47.7)	4.00 ±0.59	0.21	136 (68.0)	4.1 ±0.55	0.734
	≤1	51 (30.5)	3.88 ±0.66		38 (19.0)	4.08 ±0.48	
	2–3	27 (16.2)	4.04 ±0.47		20 (10.0)	3.96 ±0.51	
	≥4	6 (3.6)	3.54 ±0.45		6 (3.0)	4.02 ±0.05	
Exercise (per week)	No	54 (32.3)	3.89 ±0.55	0.425	46 (23.0)	4.05 ±0.52	0.954
	1–2	45 (26.9)	3.88±0.77		52 (26.0)	4.07 ±0.56	
	3–4	48 (28.7)	4.03±0.49		59 (29.5)	4.11 ±0.56	
	≥5	20 (12.0)	4.07±0.48		43 (21.5)	4.07 ±0.48	
Meal	Regular	107 (64.1)	3.94 ±0.65	0.768	175 (87.5)	4.12 ±0.53	0.011
	Irregular	60 (35.9)	3.97 ±0.47		25 (12.5)	3.83 ±0.45	
Perceived health status	Very healthy	2 (1.2)	4.42 ±0.80	0.629	9 (4.5)	4.12 ±0.35	0.043
	Healthy	50 (29.9)	4.03±0.66		57 (28.5)	4.01 ±0.59	(b<d)
	Usual	81 (48.5)	3.91±0.60		93 (46.5)	4.03 ±0.50	
	Unhealthy	30 (18.0)	3.9 ±0.45		41 (20.5)	4.28 ±0.50	
	Terribly unhealthy	4 (2.4)	3.92±0.35		0 (0.0)		
Private insurance	Yes	155 (92.8)	3.96±0.61	0.275	128 (64.0)	4.06 ±0.56	0.593
	No	12 (7.2)	3.77±0.35		72 (36.0	4.11 ±0.47	
Chronic disease	Yes	74 (44.3)	3.97 ±0.39	0.682	116 (58.0)	4.13 ±0.53	0.093
	No	93 (55.7)	3.93±0.72		84 (42.0)	4.01 ±0.55	
Reason of cancer screening	Notice	88 (52.7)	3.88±0.57	0.246	114 (57.0)	4.11 ±0.55	0.039
	Regular check up	60 (35.9)	4.00±0.64		62 (31.0)	4.04 ±0.52	(c<e)
	Checking health status	15 (9.0)	4.1±0.49		20 (10.0)	3.92 ±0.58	
	Suggestion by acquaintance	4 (2.4)	4.32 ±0.53		0 (0.0)		
	Suggestion by expert	0 (0.0)			4 (2.0)	4.71 ±0.58	
Experience of the cancer screening (For 2 years)	Yes	120 (72.5)	3.98±0.62	0.338	157 (78.5)	4.14 ±0.52	0.004
	No	47 (27.5)	3.88±0.50		43 (21.5)	3.88 ±0.52	

**Table 2:** Comparisons of Cancer Screening Knowledge, Health Beliefs, Satisfaction and Rescreening Intention

Variables	Middle-aged	Elderly	*p*
Knowledge	0.76 ±0.22	0.77 ±0.21	0.736
Health belief			
Perceived susceptibility	2.61 ±0.80	2.48 ±0.80	0.109
Perceived severity	3.98 ±0.64	3.84 ±0.74	0.037
Perceived benefit	4.27 ±0.56	4.14 ±0.48	0.019
Perceived barrier	2.58 ±0.72	2.66 ±0.73	0.272
Cancer screening satisfaction	4.42 ±0.56	4.27±0.51	0.011
Cancer screening intention	3.96 ±0.60	4.08 ±0.53	0.029

**Table 3:** Correlations of Variables with Cancer Rescreening Intention

Variables	Knowledge	Perceived susceptibility	Perceived severity	Perceived benefit	Perceived barrier	Cancer screening satisfaction
	r *(p)*					
Middle-aged						
Perceived susceptibility	0.04 (0.567)					
Perceived severity	-0.01 (0.868)	0.11 (0.174)				
Perceived benefit	-0.05 (0.463)	0.15 (0.07)	0.27 (<0.001)			
Perceived barrier	0.03 (0.716)	0.28 (<0.001)	0.27 (0.001)	-0.07 (0.363)		
Cancer screening satisfaction	0 (0.998)	0.07 (0.4)	0.03 (0.718)	0.44 (<0.001)	-0.12 (0.138)	
Cancer screening intention	0.03 (0.736)	-0.01 (0.855)	0.17 (0.03)	0.27 (<0.001)	-0.17 (0.027)	0.19 (0.016)
Elderly						
Perceived susceptibility	0.01 (0.989)					
Perceived severity	0.08 (0.28)	0.2 (0.004)				
Perceived benefit	0.21(0.003)	0.11 (0.121)	0.21(<0.001)			
Perceived barrier	-0.05 (0.53)	0.08 (0.24)	0.05 (0.472)	0.02 (0.754)		
Cancer screening satisfaction	0.24 (0.001)	-0.04 (0.57)	0.15 (0.033)	0.24 (<0.001)	-0.18 (0.01)	
Cancer screening intention	0.14 (0.044)	0.01 (0.935)	0.18 (0.01)	0.36 (<0.001)	-0.11 (0.129)	0.24 (0.001)

**Table 4:** Factors that Influence Cancer Rescreening Intention

Factors	B	S,E	β	t	p
Middle-aged					
(Constant)	2.82	0.41		6.92	<0.001
Perceived benefit	0.22	0.08	0.21	2.72	0.007
Perceived barrier	-0.17	0.06	-0.2	-2.59	0.01
Perceived severity	0.15	0.07	0.16	2.05	0.042
R^2^ =0.119 (Adjusted R^2^=0.103, F=7.30, p<0.001)					
Elderly					
(Constant)	1.92	0.37		5.18	<0.001
Perceived benefit	0.32	0.07	0.29	4.39	<0.001
Cancer screening satisfaction	0.16	0.07	0.15	2.27	0.024
Experience of the cancer screening (Yes)	0.18	0.08	0.14	2.09	0.038
Reason of cancer screening (Suggestion by expert)	0.51	0.24	0.13	2.09	0.038
R^2^ =0.192 (Adjusted R^2^=0.176, F=11.62, p<0.001)					

## Discussion

This study was conducted to identify the current status of cancer screening among middle-aged and elderly and compare the factors that influence re-screening intention in order to obtain foundational data for establishing a cancer screening strategy suited for middle-aged and elderly. Results showed that re-screening intention was slightly higher in elderly participants, with mean scores of 3.96 for middle-aged and 4.08 for elderly subjects, respectively. This was lower than the mean score of 4.24 in a previous study conducted among women in their 50s (Yoo, 2005). The middle-aged group is socially active and maintains physical vigor and health compared to the elderly; this can be interpreted as the latter being less interested in health and having a slightly passive attitude toward taking action. 

There was no statistically significant difference in cancer re-screening intention according to subject characteristics in the middle-aged group. This was inconsistent with the findings of several studies that reported screening rates and intentions associated with healthy behaviors (e.g., smoking, exercise, diet), employment, and education (Park et al., 2015, Tolma et al., 2014). Conversely, the re-screening intention of elderly persons was higher among subjects who had regular meals, perceived health condition was not good, had been recommended for screening by an expert, and had experience with cancer screening. This is similar to several studies wherein smoking, drinking, exercise, disease, medical history, recommendations from medical personnel, and screening experience impacted cancer screening (Martires et al., 2014; Lee, 2019).

Differences were found in the health beliefs of middle-aged and elderly subjects in perceived severity and perceived benefits, with middle-aged subjects scoring higher for perceived severity and benefits. This may have been because most of the elderly participants had chronic diseases, leading them to think that cancer screening would not be as effective. Despite having higher perceived severity and benefits than the elderly, re-screening intention was low among middle-aged subjects, indicating that even when they were aware of the necessity of cancer screening, they were not translated into action. Therefore, it is necessary to find a way to increase interest in cancer screening and enable middle-aged people to undergo cancer screening.

This study attempted to identify and compare the factors influencing re-screening intention among middle-aged and elderly from various dimensions. The factors that influence cancer re-screening intention among middle-aged persons were perceived benefits, perceived barriers, and perceived severity of cancer screening. Cancer re-screening intention was higher in those with high perceived benefits, low perceived barriers, and high perceived severity was high. For the middle-aged participants, general characteristics or health behaviors did not affect cancer screening intention, and attitudes toward cancer screening were more influential. Cullati et al., (2009) reported that attitude played a more significant role than demographic variables in cancer screening among middle-aged individuals. An (2014) reported differences according to perceived susceptibility, perceived severity, perceived significance, and perceived barriers; similarly, Kim (2015a) reported that positive behavioral attitudes and higher levels of subjective norms were correlated with higher screening intention. 

For middle-aged individuals, perceived severity and perceived benefits both ranked high among health beliefs, indicating they had difficulty taking action due to barriers despite being aware of the need for cancer screening. As such, conditions that ease participation in cancer screening must be created. To increase the screening rate in middle-aged who lack time due to work, various methods must be considered, such as diversifying the hours for screening, a postal test system, or screening during weekends or after work hours. 

The factors related to re-screening intention among the elderly were perceived benefits, satisfaction, cancer screening experience, and the reason for cancer screening. Cancer re-screening intention was higher in those with high perceived benefits, high cancer screening satisfaction, experience with cancer screening, and screening recommended by an expert. While the attitude toward cancer screening was found to be the major factor influencing middle-aged persons in this study, the factors affecting elderly were satisfaction with, experience with, and reason for cancer screening, rather than behavioral factors. 

This study showed that a higher level of satisfaction with cancer screening was correlated with higher re-screening intentions among elderly, a finding consistent with the results of Kim (2015a) and Madlensky et al., (2003), who reported that cancer screening satisfaction affected re-screening intention. To increase the re-screening rate among the elderly, it is, therefore, necessary to increase the level of satisfaction with cancer screening. In a previous study (Madlensky et al., 2003), the inconvenience of the procedures was found to pose the greatest barriers to cancer screening. Specific methods to increase the convenience of the procedures must be developed. In addition, a low level of knowledge or literacy may also be a problem among the elderly, thus screening strategies that increase satisfaction by providing customized information and reminder systems that easily and effectively deliver information must be established.

In this study, the re-screening intention was higher for elderly with experience with cancer screening, consistent with findings of previous studies (Kim et al., 2010; Lee, 2019) that reported that experience with cancer screening influences screening intention or had a higher screening rate. This indicates that, due to the need for periodic repetition of cancer screening, elderly who already have experience with cancer screening are more likely to continue screening. Efficient and continuous cancer screening can thus be achieved once new subjects are motivated to undergo screening. 

Among factors influencing cancer re-screening in elderly persons, a recommendation for cancer screening from an expert led to a higher re-screening intention, consistent with studies that showed that 54.1% of participants preferred to be recommended by family members, friends, or neighbors (Kye and Moon, 2010). Specifically, a study showed that recommendation by a doctor was highly influential for cancer screening among elderly and also affected re-screening intention (Park and Park, 2014) thus a recommendation from a doctor may be an effective strategy to increase re-screening intention among elderly. A social support program to promote cancer screening may have to be developed. 

The current national cancer screening is available for those aged 40 years and older, regardless of the subject group, thus customized strategies considering age demographics are needed to encourage people to undergo general health and cancer screening programs, especially cancer screening programs for elderly individuals. In conclusion, factors influencing cancer re-screening intentions differed between middle-aged and elderly. Therefore, a customized and systemic approach toward cancer screening programs is suggested to promote continuous screening considering the characteristics of middle-aged and elderly. 

This study compared the differences in the factors that influence cancer re-screening intention in middle-aged and elderly in Korea and may provide basic data for developing a strategy customized for each age group. This study has several limitations, including sample collection and distribution, with the samples having been collected from regional communities and distribution being uneven due to convenience sampling.

## Author Contribution Statement

Hee Jung Kim: Conceptualization, Methodology, Validation, Investigation, Writing-Original Draft preparation, Writing-Review and Editing.

## Availability of data (if apply to your research)

The datasets generated during the current study are available from the corresponding author on reasonable request.
